# Supplementary material for: Centella asiatica-Derived Endothelial Paracrine Restores Epithelial Barrier Dysfunction in Radiation-Induced Enteritis
Source: Cells. 2022 Aug 16;11(16):2544. doi: 10.3390/cells11162544 (PMC9406831; doi:10.3390/cells11162544)
Supplement: Supplementary file 1 [file cells-11-02544-s001.zip › cells-1818616-supplementary.pdf]

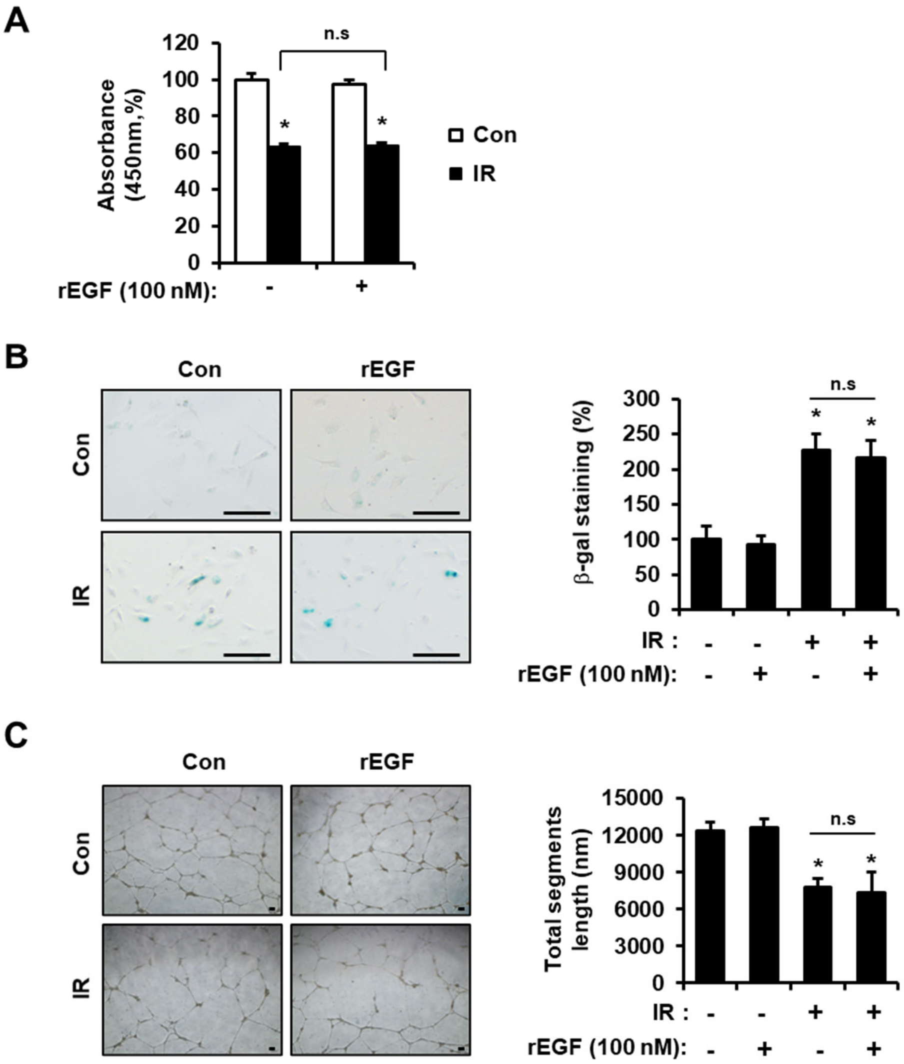

Figure S1. Epidermal growth factor does not influence repair of radiation-induced endothelial cell damage. (**A**) Viability of HUVECs after treatment with or without recombinant epidermal growth factor (rEGF; 100 nM). The effect of rEGF on HUVEC viability was assessed by CCK-8 assay. Bars represent the percentage of proliferated cells normalized to that of the corresponding control. (**B**) Determination of senescent activity by rEGF. HUVECs were irradiated and treated with or without rEGF. Senescent HUVECs were quantified by performing a◎◎-galactosidase assay. Quantified senescent HUVECs were plotted as a bar graph (right). (**C**) Determination of angiogenic activity. HUVECs were re-seeded onto Matrigel-coated wells in the presence or absence of rEGF. Total segments length per five fields were quantified and plotted as a bar graph (right). Data are presented as the means ± standard deviation of the mean. Images are representative of 3 independent experiments. *p <0.05 vs. negative control (Con). #p < 0.05 vs. irradiated control (IR). Scale bar = 100 ◎m.

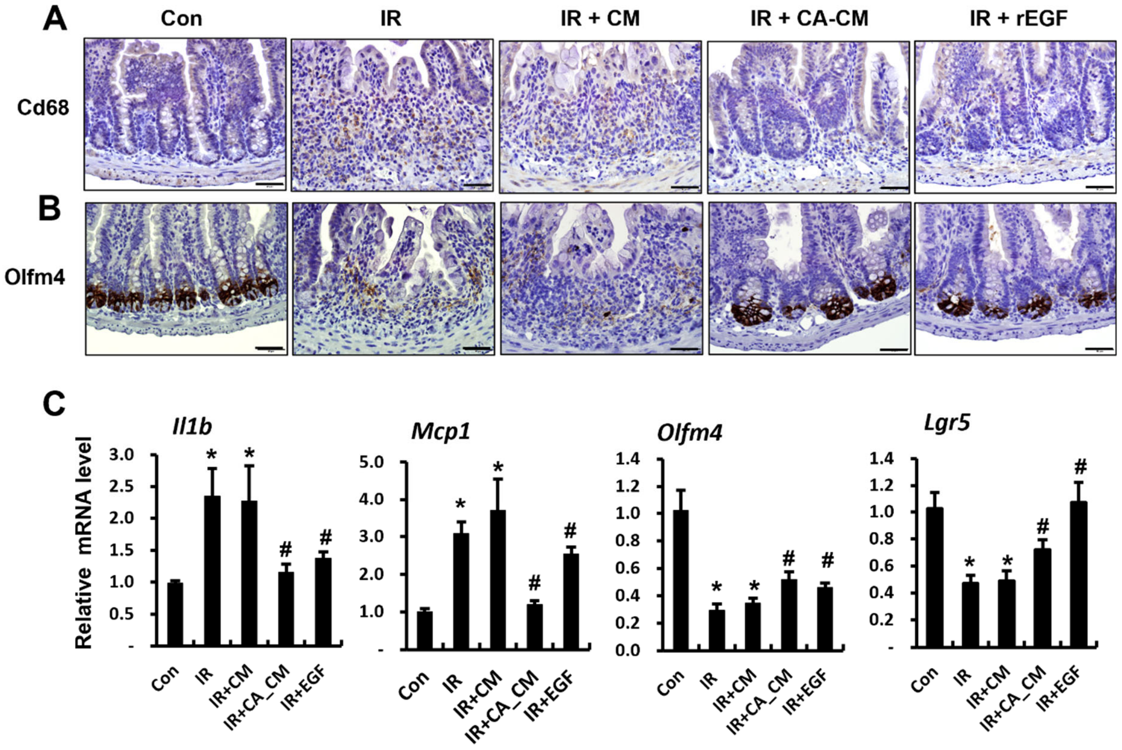

Figure S2. Centella asiatica-derived endothelial epidermal growth factor inhibits radiation-induced inflammation and improves stem cell property. Mouse groups are as follow; Control (Con), irradiated (IR), irradiated mouse administrated with the conditioned media (CM) of irradiated HUVECs (IR + CM), CM of Centella asiatica (CA)-treated irradiated HUVECs (IR + CA-CM), and recombinant EGF (IR + rEGF). Representative images of the small intestine stained with (**A**) Cd68 and (**B**) Olfm4. Scale bars represent 50◎◎m. (**C**) mRNA levels of *Il1b*, *Mcp1*, *Lgr5*, and *Olfm4* in small intestine of Con, IR, IR + CM, IR + CA-CM, and IR + rEGF group. Data are presented as the mean ± standard error of the mean; n = 6 mice per group. *P < 0.05 compared to the Con group; #P < 0.05 compared to the IR group. Scale bars represent 100 ◎m. All data represent at least two independent experiments.
